# Notes and News

**Published:** 1900-10-13

**Authors:** 


### Notes and News.

On October 30th Lord Lister will open the new scientific laboratories at King's College.

Mr. M. Cursham Corner has been appointed a Justice of the Peace for the County of London, Tower Division.

A conference of the representatives of each Poor-Law authority in the Metropolis has been appointed to be held in the St. Martin's Town Hall on Monday next, the 15th inst., at 3 p.m., to consider the question of establishing hospitals for the open-air treatment of consumption.

The management of the Poplar and Stepney Sick Asylum approached the Local Government Board some time ago with a view to securing an establishment for their district. The Local Government Board expressed an opinion that such an establishment should serve the whole Metropolis and be under a metropolitan authority, and to further such a scheme suggested such a conference as is to be held on the 15th.

Another patient has died from the plague in Glasgow, but no new cases are reported. The process adopted by the Glasgow Health Authorities in infected quarters has been as follows:—All walls, ashpits, closes, and passages in the infected district are limewashed, with an addition of chloride of lime in the proportion of 1 in 100. Streets and courts are washed with a hypochlorite of soda solution (chloros) in the proportion of 1 in 30. The walls, floors, and ceilings of rooms are disinfected—first, with sulphur, and afterwards with formalin.

The establishment of sanatoria for tuberculosis in France will not be left to local or individual effort, as a number of the medical profession in Paris are forming a company to promote popular institutions for the purpose. The capital with which it is intended to commence operations is remarkably small, only 300,000 francs, so that progress must be necessarily slow, even if each establishment proves profitable. American authorities are looking far ahead, and endeavouring to provide facilities for future generations of the tuberculous. The Forestry Commission of New York State intend rearing forests with the idea that in the future the care of consumptives will be undertaken by the State.

The first woman doctor for prison work in Germany has been appointed in the person of Dr. Agnes Hacker, who will act as Medical Officer to female prisoners in Berlin.

At St. Thomas's Hospital Medical School an Entrance Scholarship in Natural Science for £150 has been awarded to Mr. George Young Worrall, and the University Scholarship in Anatomy and Physiology for £50 has been awarded to Mr. Walter L. Harnett, B.A. Cantab.

The following scholarships and exhibitions have been awarded at University College, London:—Medical Entrance Scholarships: Bucknill Scholarship, £30 a year for five years, and Fifty-five Guineas Scholarship (equally divided), H. T. Mant and W. S. Sweet; Fifty-five Guineas Scholarship, H. E. Dyson. Medical Exhibitions: 76 guineas each, C. W. Forsyth and A. M. H. Gray.

The Derry Infirmary is practically a rate-supported institution, seeing that it draws its income of £1,800 or so, all but £300 or £400, from the rates. In spite of this its medical staff is an honorary one. It is further suggested that the appointments should only be held for three years. It cannot be wondered at that some of the medical men of Derry are not satisfied with the use to which the medical profession is being put.

A new isolation hospital has recently been opened at Leicester. It stands on a site of fourteen acres. The administration block is a two-storeyed building, the wards mostly being situated in one-floor pavilions. Four pavilions with twenty-six beds in each are devoted to scarlet-fever cases. A pavilion of twenty-eight beds is for typhoid, and two pavilions for twenty-eight beds are for observation cases. The pavilions are not connected in any way. They are mainly divided into two large wards and one small ward. The hospital is lighted by electricity, and the fittings, heating, and ventilating arrangements have been carefully thought out. The Town Council of Leicester originally voted £54,000 towards the establishment, but it is expected that the sum expended will exceed this.

Owing to the prevalence of enteric fever in London the managers of the Metropolitan Asylums Board have resumed the occupation of the 36 beds which they hired last year at the Metropolitan Hospital. These beds will be taken for three months, and paid for at the rate of £50 per week, this payment, it is stated, including, as before, medical attendance, nursing, and domestic staff, the Asylums Board managers undertaking the laundry. The arrangement is one which we have no doubt will be found very satisfactory both by the hospital and by the Asylums Board. We cannot, however, shut our eyes to the fact that such a farming out of the services of the medical staff raises serious questions of medical ethics. It cannot be right that the medical staff of this hospital should give their gratuitous services to a great spending department like the Metropolitan Asylums Board, a public body which is supported by the rates and is literally rolling in wealth, if one may judge by the money spent by them in the erection of hospitals. If, on the other hand, it is maintained that the sum charged covers the proper fees payable to these physicians for the treatment of these patients, then it is a serious question whether it is a right thing for a hospital to sell to a rich public body the services which its medical officers give to it as a charity.

